# Lung ultrasound score predicts outcomes in COVID-19 patients admitted to the emergency department

**DOI:** 10.1186/s13613-020-00799-w

**Published:** 2021-01-11

**Authors:** Julio Cesar Garcia de Alencar, Julio Flavio Meirelles Marchini, Lucas Oliveira Marino, Sabrina Correa da Costa Ribeiro, Cauê Gasparotto Bueno, Victor Paro da Cunha, Felippe Lazar Neto, Rodrigo Antonio Brandão Neto, Heraldo Possolo Souza, the COVID U. S. P. Registry Team, Fernando Salvetti Valente, Hassan Rahhal, Juliana Batista Rodrigues Pereira, Eduardo Messias Hirano Padrão, Annelise Passos Bispos Wanderley, Bruno Marques, Felipe Liger Moreira, Luz Marina Gomez Gomez, Millena Gomes Pinheiro Costa, Lucas de Oliveira Utiyama, Felipe Mouzo Bortoleto, Renan Dourado Tinel, Gabriel Martinez, Saionara Maria Nunes Nascimento, Lucas Gonçalves Dias Barreto, Karina Turaça, Debora Lopes Emerenciano, Daniel Rodrigues Ribeiro, Danilo Dias de Francesco, Eduardo Mariani Pires de Campos, Stefany Franhan Barbosa de Souza, Geovane Wiebelling da Silva, Andrew Araujo Tavares, Clara Carvalho de Alves Pereira, Ademar Lima Simões, Gustavo Biz Martins, Leonardo Antonio Coimbra Moreira, Maria Lorraine Silva de Rosa, Pedro Henrique Rodrigues Santana, Thiago Areas Lisboa Netto, Eduardo Padula, Julio Cesar Leite Fortes, Mauricio Ursoline do Nascimento, Rafael Faria Pisciolaro, Rodolfo Affonso Xavier, Marcel Yukio Kamonseki, Patricia Perez Barroso, Rodolfo Avelino de Souza, Yago Henrique Padovan Chio, Edwin Albert D’Souza, Arthur Petrillo Bellintani, Rodrigo Cezar Miléo, Rodrigo Werner Toccoli, Fernanda Máximo Fonseca e Silva, João Martelleto Baptista, Marcelo de Oliveira Silva, Giovanna Babikian Costa, Rafael Berenguer Luna, Henrique Tibucheski dos Santos, Mariana Mendes Gonçalves Cimatti De Calasans, Marcelo Petrof Sanches, Diego Juniti Takamune, Luiza Boscolo, Pedro Antonio Araújo Simões, Manuela Cristina Adsuara Pandolfi, Beatriz Larios Fantinatti, Gabriel Travessini, Matheus Finardi Lima de Faria, Ligia Trombetta Lima, Bianca Ruiz Nicolao, Gabriel de Paula Maroni Escudeiro, João Pedro Afonso Nascimento, Bruna Tolentino Caldeira, Laura de Góes Campos, Vitor Macedo Brito Medeiros, Tales Cabral Monsalvarga, Isabela Harumi Omori, Diogo Visconti Guidotte, Alexandre Lemos Bortolotto, Rodrigo de Souza Abreu, Nilo Arthur Bezerra Martins, Carlos Eduardo Umehara Juck

**Affiliations:** 1 Emergency Department, Hospital das Clínicas da Faculdade de Medicina da Universidade de São Paulo, 255, Dr. Enéas de Carvalho Aguiar st., São Paulo, SP Brazil; 2 Medical student, Faculdade de Medicina da Universidade de São Paulo, São Paulo, Brazil

**Keywords:** COVID-19, Severe acute respiratory syndrome coronavirus 2, Ultrasonography, Critical care, Emergency medicine

## Abstract

**Background:**

During the COVID-19 pandemic, creating tools to assess disease severity is one of the most important aspects of reducing the burden on emergency departments. Lung ultrasound has a high accuracy for the diagnosis of pulmonary diseases; however, there are few prospective studies demonstrating that lung ultrasound can predict outcomes in COVID-19 patients. We hypothesized that lung ultrasound score (LUS) at hospital admission could predict outcomes of COVID-19 patients. This is a prospective cohort study conducted from 14 March through 6 May 2020 in the emergency department (ED) of an urban, academic, level I trauma center. Patients aged 18 years and older and admitted to the ED with confirmed COVID-19 were considered eligible. Emergency physicians performed lung ultrasounds and calculated LUS, which was tested for correlation with outcomes. This protocol was approved by the local Ethics Committee number 3.990.817 (CAAE: 30417520.0.0000.0068).

**Results:**

The primary endpoint was death from any cause. The secondary endpoints were ICU admission and endotracheal intubation for respiratory failure. Among 180 patients with confirmed COVID-19 who were enrolled (mean age, 60 years; 105 male), the average LUS was 18.7 ± 6.8. LUS correlated with findings from chest CT and could predict the estimated extent of parenchymal involvement (mean LUS with < 50% involvement on chest CT, 15 ± 6.7 vs. 21 ± 6.0 with > 50% involvement, *p* < 0.001), death (AUC 0.72, OR 1.13, 95% CI 1.07 to 1.21; p < 0.001), endotracheal intubation (AUC 0.76, OR 1.17, 95% CI 1.09 to 1.26; *p* < 0.001), and ICU admission (AUC: 0.71, OR 1.14, 95% CI 1.07 to 1.21; *p* < 0.001).

**Conclusions:**

In COVID-19 patients admitted in ED, LUS was a good predictor of death, ICU admission, and endotracheal intubation.

## Background

The novel coronavirus disease 2019 (COVID-19) poses an immense and urgent threat to global health [1]. The entire world is witnessing health care systems, and emergency departments in particular, being overwhelmed by the COVID-19 pandemic [2]. Adequately managing available resources may be the key point to overcoming the surge of patients and saving lives [3]. In this context, tools to assess disease severity and prognosis in COVID-19 patients are one of the most important assets in reducing the burden on emergency departments.

Symptoms of COVID-19 vary widely, from asymptomatic disease to severe pneumonia with life-threatening complications [4]. Severe illness usually begins approximately one week after the onset of symptoms, and a striking feature of COVID-19 is the rapid progression to respiratory failure [5]. Patients with severe COVID-19 commonly meet the criteria for acute respiratory distress syndrome (ARDS), which is defined as the acute onset of bilateral infiltrates, severe hypoxemia, and lung edema that is not fully explained by cardiac failure or fluid overload [6]. Even though it can meet the ARDS Berlin definition, COVID-19 pneumonia is a specific disease with peculiar phenotypes, and some investigators propose the presence of two types of patients (“non-ARDS” or type 1, and ARDS or type 2) with different pathophysiologies, distinguishable by chest computed tomography (CT) [7].

Because most patients with severe COVID-19 have pneumonia, imaging is particularly useful for diagnosis and possibly to predict adverse outcomes [8]. Unfortunately, there are downsides. Chest radiography is not sensitive for COVID-19 and usually shows no abnormal findings in the early stages of infection [9]. Chest CT detects early COVID-19 pneumonia with high sensitivity, and small studies have suggested that it can be used to assess disease severity and guide clinical management [10, 11]. However, obtaining a CT scan requires transporting critically ill patients [12], exposes the patient to radiation [13], and demands that rigorous infection control procedures be followed before scanning subsequent patients [14]. Moreover, CT equipment is not widely available, especially in developing countries [15].

Lung ultrasound is widely used in emergency departments because it is user-friendly, broadly available, low-cost, and has a high accuracy for diagnosing pulmonary diseases [16]. Recent reports suggest that, in COVID-19 patients, lung ultrasound could be useful in several scenarios: to quantify the severity of lung involvement in periodic assessments, to look for findings suggestive of pneumonia, and to monitor the dynamic effects of mechanical ventilation and recruitment maneuvers on lung aeration [3, 17].

The lung ultrasound score (LUS) is a semiquantitative score that measures lung aeration loss caused by different pathological conditions [18, 19]. There are a few prospective studies demonstrating that lung ultrasound can predict outcomes in COVID-19. LUS has been strongly correlated with pulmonary involvement and provides risk stratification, including prediction of need for mechanical ventilation and mortality [20, 21].

Within this context, we hypothesized that LUS at hospital admission could predict outcomes in patients with COVID-19.

## Methods

### The aim, design and setting

This prospective cohort study was conducted from 14 March through 6 May 2020 in the emergency department (ED) of Hospital das Clinicas da Universidade de São Paulo (HC-FMUSP), a 2200-bed urban, academic medical center comprising five institutes and two auxiliary hospitals. During the pandemic, the HC-FMUSP ED has been designated exclusively for the reception and care of patients with COVID-19.

The primary endpoint of the study was death from any cause by 20 July 2020. The secondary endpoints were any ICU admission and endotracheal intubation for respiratory failure by 20 July 2020. We chose this date solely to expedite the communication of our findings.

The study protocol was approved by the local Ethics Committee (opinion number 3.990.817; CAAE: 30417520.0.0000.0068), which also waived the need for written informed consent. The present report adheres to the STROBE guidelines.

### Patients

Patients aged 18 years and older who were admitted to the ED with suspected or confirmed COVID-19 were considered eligible. Patients who had advance directives (do not intubate or do not resuscitate) and pregnant women were excluded.

Patients who did not test positive for COVID-19 by reverse-transcriptase polymerase chain reaction (RT-PCR) assay of nasopharyngeal swab or tracheal aspiration specimens were also excluded. For the intubation and ICU admission outcomes we excluded patients who were already intubated before we performed lung ultrasound (We still included these patients in mortality analysis).

### Research protocol

After selection, patients were asked for permission to be included in the study. Once permission was granted, a researcher interviewed the patient and collected data through a standardized form using the TeamScope® software (TeamScope Holding Limited, London, England). The following variables were collected: age, sex, day of illness, date of admission, and signs and symptoms on admission.

A second blinded investigator who was not involved in patient care performed the lung ultrasound. Due to the investigators’ limited availability, scans were performed only from Monday through Thursday, from 8:00 a.m. to 8:00 p.m. The investigators were aware of the presenting symptoms and the most visible physical signs but were blinded to all other clinical information including the radiologic findings.

Subsequently, a third investigator prospectively completed a second questionnaire using the RedCap® software (Vanderbilt University, Nashville, Tennessee, USA) with the following variables collected from electronic medical records: chest CT findings, hospitalization outcome (including hospital discharge and death), need for ICU referral, and need for invasive mechanical ventilation.

Chest CT was performed only for clinical purposes independent of the study protocol. Blinded attending radiologists reported chest CTs as consistent or inconsistent with the most typical pattern described in COVID-19, which includes ground-glass opacities, sometimes with superimposed interlobular septal thickening (crazy paving), consolidations and reversed halo, presenting a bilateral multilobar distribution, predominantly peripheral, with mild predilection for the posterior regions and lower lobes, and gave a visual estimate of the extent of parenchymal involvement (greater or less than 50%) [10].

### LUS protocol

The investigators were four emergency medicine attending physicians with at least 5 years’ experience in point-of-care emergency ultrasonography.

The patient was preferably examined in the sitting position. When this position could not be maintained due to clinical deterioration or poor compliance, the examination was performed in the supine or semirecumbent position. The posterior lung fields were scanned in the sitting position or, when not feasible, by turning the patient onto lateral decubitus position on both sides successively.

We performed lung ultrasound with a Sonosite Edge II portable ultrasound system and a 2- to 5-MHz convex transducer. The examination should start by adjusting the machine to abdominal pre-set to a depth of 15 cm, and the focus should be adjusted to the area of interest. The probe was placed vertically perpendicular to the ribs. Each point was examined for at least one complete respiratory cycle.

The LUS protocol involves the examination of 12 lung regions, performed in around than five minutes in our service: the upper and lower parts of the anterior, lateral, and posterior aspects of the left and right chest wall. Each region is scored according to four ultrasound aeration patterns. For a given region of interest, we allocated points according to the worst ultrasound pattern observed. The final LUS is the sum of points in all 12 regions and ranges from 0 to 36 [3, 22, 23]. In our study, some terms of the LUS were modified, inspired from Lichtenstein’s nomenclature: 0 points—presence of lung sliding with A lines or one or two isolated B lines; 1 point—moderate loss of lung aeration with three or four B lines (septal rockets); 2 points—severe loss of lung aeration with five or more B lines (glass rockets); and 3 points—presence of a hypoechoic poorly defined tissue characterized by complete loss of lung aeration (consolidation) [24].

### Statistical analyses

Data are presented as percentages for categorical variables and the mean ± standard deviations for continuous variables. All data were tested for normality using the Kolmogorov–Smirnov test. When distribution was normal, we used a two-tailed Student’s t-test. We performed logistic regression to explore the associations of LUS with intubation, ICU admission, and mortality. We calculated the area under the receiver operating characteristic curve (AUC) for each regression and accepted statistical significance at *p* ≤ 0.05. All analyses were performed using Stata 13 software (College Station, TX, USA).

## Results

### Patients

**Fig. 1:**
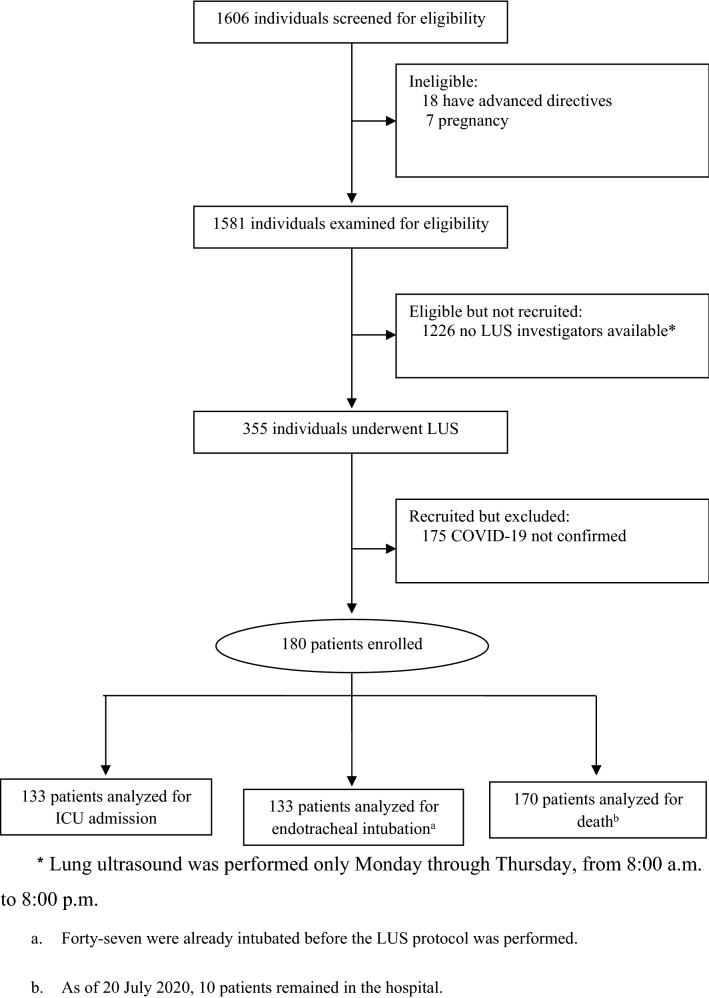
Diagram of patient flow through the study

During the study period, we admitted 1606 consecutive patients. Of these, 506 patients confirmed COVID-19 by RT-PCR, and 180 were enrolled (Fig. 1). The median age was 60 years, and 105 patients (58%) were men. The clinical and laboratory characteristics of the patients are summarized in Table 1.

**Table 1:** Baseline characteristics of patients, vital signs, and laboratory results at admission

Characteristic (N = 180)	Median (IQR) or N (%)
Age (y)	60 (49–70)
Sex	
Female	75 (42%)
Male	105 (58%)
Comorbidities	
Hypertension	98 (55%)
Diabetes	69 (39%)
Congestive heart failure	25 (14%)
Chronic respiratory disease	15 (8%)
Chronic and end-stage renal disease	11 (6%)
Duration of symptoms before hospital admission (days)	7 (5–10)
Oxygen saturation < 93%^b^	78 (43%)
Oxygen saturation—Median (IQR)	93 (90–96)
Received supplemental oxygen at triage	136 (76%)
Type of supplemental oxygen	
Nasal cannula	53 (30%)
Nasal cannula oxygen flow	2.8 ± 1.4 L O_2_/min
Venturi mask (FiO_2_ = 50%)	1 (1%)
Nonrebreather mask	35 (19%)
Nonrebreather mask oxygen flow	11.9 ± 3.5 L O_2_/min
Mechanical ventilation	47 (27%)
Mechanical ventilation FiO_2_	86.5 ± 21.8%
Respiratory rate > 24 breaths/min^b^	91 (51%)
Respiratory rate—median (IQR)	25 (20–30)
Heart rate > 100 beats/min	53 (29%)
Heart rate—median (IQR)	92 (80–103)
PaO_2_/FiO_2_ on admission, median (IQR)	120 (64–230)
LUS^a^	18.7 ± 6.8

*IQR* interquartile range, *LUS* lung ultrasound score

^a^Mean ± standard deviation

^b^Most of these patients did not tolerate removal of supplemental oxygen to measure at room air

### Outcomes

All patients underwent LUS on the day of emergency department admission. The average LUS was 18.7, with a standard deviation of 6.8.

We enrolled 180 patients, 109 (60%) were discharged alive, 61 (33%) died, and 10 patients (5%) were still in the hospital at the study endpoint. As of 20 July 2020, 74 patients (56%) had been treated in the ICU, and 52 (39%) received invasive mechanical ventilation. Forty-seven patients were already intubated at admission or were intubated shortly after admission, before lung ultrasound could be performed, and were excluded from intubation and ICU analysis. The mean time between lung ultrasound and intubation was 2.1 ± 1.9 days with a median of 2 days.

Among the 142 patients who underwent chest CT on admission, LUS was associated with the extension of COVID-19 pneumonia on CT. The mean LUS in patients with < 50% involvement on chest CT was 15 ± 6.7 vs. 21 ± 6.0 in those with > 50% involvement (*p* < 0.001).

Duration of symptoms before admission did not correlate with LUS. We also performed a univariate analysis with symptoms at admission and laboratory tests and found no correlation with mortality in our patients. However, age and bilateral lung involvement > 50% on chest CT were predictors of death.

**Fig. 2:**
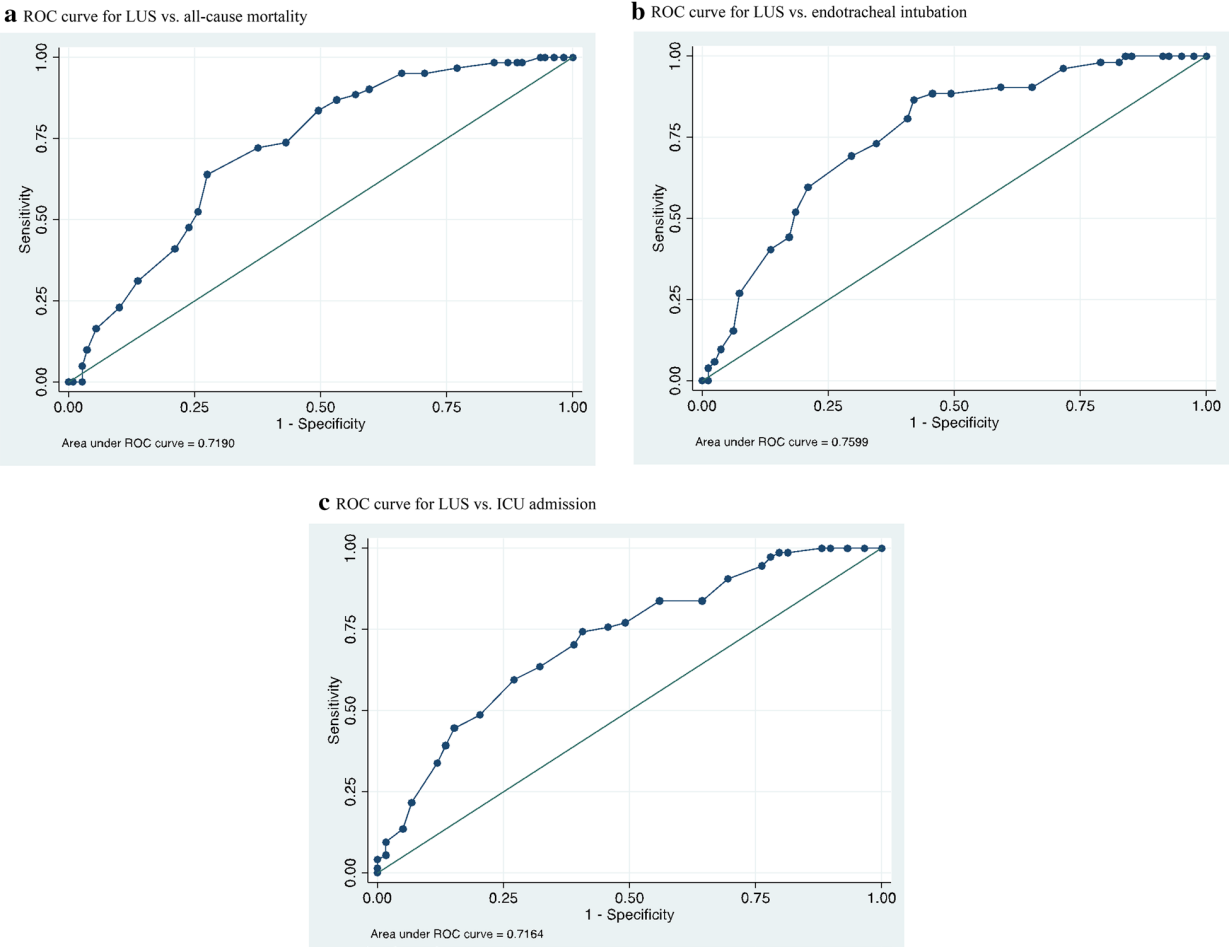
ROC Curves and Outcomes. **a** For LUS versus all-cause mortality. **b** For LUS versus endotracheal intubation. **c** For LUS versus ICU admission

As observed in Fig. 2 and Table 2, LUS could predict death, endotracheal intubation, and ICU admission. We plotted AUC to define useful cutoffs for LUS, as shown in Table 3.

**Table 2:** LUS and outcomes in patients with COVID-19

	Patients	LUS (mean ± SD)	OR (95% CI)	p**
Primary outcome				
Deceased	61/170 (36%)	21.6 ± 4.9	1.13 (1.07–1.21)*	< 0.001
Discharged alive	109/170 (64%)	16.7 ± 4.9		
Secondary outcomes				
Intubated	52/133 (39%)	21.3 ± 4.9	1.17 (1.09–1.26)	< 0.001
Not intubated	81/133 (61%)	15.2 ± 7.1		
Admitted to ICU	74/133 (56%)	20.0 ± 5.9	1.14 (1.07–1.21)	< 0.001

^*^Adjustment for age did not change results

^**^p-values calculated by Student’s t-test

**Table 3:** LUS and outcome cutoffs

	LUS cutoff	Sensitivity	Specificity
Discharged alive	< 16	40%	90%
Deceased	≥ 26	23%	90%
Not intubated during hospital stay	< 15	41%	90%
Intubated during hospital stay	≥ 25	27%	93%
Not admitted to ICU	< 13	31%	91%
Admitted to ICU	≥ 25	22%	93%

## Discussion

The COVID-19 pandemic has brought numerous patients complaining of fever, cough, and dyspnea to ED around the world. Proper assessment of the severity and extent of pulmonary involvement is of paramount importance to select patients who will be admitted to hospital wards or ICUs and thus ensure adequate management of overwhelmed healthcare resources.

In this present study, we analyzed the prognostic value of lung ultrasound in ED COVID-19 patients. We also described a useful tool for lung ultrasound findings that can be summarized in a simple ordinal scoring system (LUS) which was able to discriminate patients’ outcomes.

The findings in this study significantly correlate clinical severity of COVID-19 pneumonia and extent of lung pathology detected by LUS, suggesting the utility of LUS in risk stratification of COVID-19 patients and clinical decision-making. The use of LUS to quantify and monitor changes in lung aeration has been described in critically ill patients with ARDS [22]. In COVID-19 patients, contrary to what has been described in ARDS, interstitial patterns and consolidations contribute almost equally to lack of aeration, thus, the severity of respiratory impairment seems to be related to the overall proportion of lung tissue showing ground-glass opacities [7]. Furthermore, the peripheral distribution of lung infiltrates in COVID-19 makes lung ultrasound a reliable imaging study [20].

In our study, LUS had a good level of discrimination between admitted patients (including intubation and dead), and increased LUS was associated with worsening disease. LUS predicts mortality with AUC 0.72, and score ≥ 26 had 90% specificity for mortality during admission. Interestingly, two recent studies also demonstrated that LUS has a good agreement in the assessment of outcomes in COVID-19 patients: Brahier et al. and Youden et al. showed correlation between LUS and mortality with AUC 0.76 and 0.78, respectively [20, 21].

Lung ultrasound can dynamically assess the ventilation status and provided earlier prediction of pulmonary ventilation status and disease deterioration [22]. In our study, LUS also increased progressively according to clinical severity, and LUS of the intubated was higher than that of the non-intubated group. LUS ≥ 25 on admission had 90% specificity for needed intubation, and may be a warning for intubation or exacerbations in critically ill COVID-19 patients in ED.

Moreover, we describe a significant relationship between extent of lung pathology detected by LUS and chest CT. This finding demonstrates that lung ultrasound is a viable instrument, easily performed at the bedside, to evaluate pneumonia severity in COVID-19 patients.

Some limitations of this study must be addressed. First, it was a single-center study conducted at a large academic hospital in São Paulo, Brazil. Second, the level of expertise required to detect small changes in LUS, together with operator dependence, may limit the clinical applicability of lung ultrasound. Third, although lung ultrasound is operator-dependent, we did not test an inter intra-observer agreement. To minimize these limitations, experienced physicians performed LUS using a standardized procedure and a pre-defined scoring method. Fourth, the absence of data on patients who remained hospitalized at the date of final data collection may has biased the findings. It would have been better to wait for all patients to achieve a definite outcome; however, because our results are already significant and we believe they are relevant, we chose to sacrifice these data for the sake of reporting our findings more quickly. Lastly, some authors do not use the LUS and replace it by short but quantitative descriptions of ultrasound disorders [24].

## Conclusions

In this study, despite some limitations, LUS was a good predictor of death, ICU admission, and endotracheal intubation in patients with COVID-19 admitted in ED. This finding can help emergency physicians determine rapidly the patient’s disposition. The study provides support for further research, ideally combining clinical, laboratory, and imaging parameters, to estimate the risk of poor outcomes from COVID-19 infection.

## Data Availability

The data that support the findings of this study are available from the corresponding author JCGA upon reasonable request.

## Abbreviations

ARDS: Acute respiratory distress syndrome
AUC: Area under the ROC curve
CI: Confidence interval
COVID 19: Coronavirus disease 2019
CT: Computed tomography
ED: Emergency department
ICU: Intensive care unit
IL-6: Interleukin-6
LUS: Lung ultrasound score
RT-PCR: Reverse-transcriptase polymerase chain reaction
